# Correspondence

**Published:** 1900-04

**Authors:** Isadore Dyer

**Affiliations:** Secretary New Orleans Polyclinic.


The report has been circulated that the New Orleans Polyclinic has been suspended on account of smallpox in New Orleans. This statement is absolutely false. The Polyclinic has had a continuous course since November 20, and will continue until May 12. The smallpox situation in New Orleans has at no time justified any apprehension on the part of students attending the Polyclinic or on the part of those who might wish to do so.

ISADORE DYER, M.D.

February 24, 1900. Secretary New Orleans Polyclinic.

